# Enhancement of Femtosecond Photon Echo Signals From an Inhomogeneously Broadened InAs Quantum Dot Ensemble Using Chirped Pulses

**DOI:** 10.1002/nap2.70013

**Published:** 2026-02-05

**Authors:** Yuta Kochi, Yutaro Kinoshita, Masanari Watanabe, Ryutaro Ide, Kouichi Akahane, Junko Ishi‐Hayase

**Affiliations:** ^1^ School of Fundamental Science and Technology Keio University Yokohama Kanagawa Japan; ^2^ Center for Spintronics Research Network Keio University Yokohama Kanagawa Japan; ^3^ National Institute of Information and Communications Technology Koganei Tokyo Japan

**Keywords:** coherent control, nonlinear optics, quantum dot

## Abstract

Photon echo (PE) techniques offer a promising approach to optical quantum memory, yet their implementation in conventional platforms, such as rare‐earth‐ion‐doped crystals, is hindered by limited bandwidths. Semiconductor quantum dot (QD) ensembles, featuring THz‐scale inhomogeneous broadening and sub‐picosecond dynamics, provide an attractive alternative for ultrafast applications. However, achieving coherent control across such broad spectral ranges remains challenging due to detuning and spatial field inhomogeneities, which reduce PE efficiency. In this work, we demonstrate that chirped rephasing pulses satisfying adiabatic conditions enable robust adiabatic rapid passage (ARP) across an inhomogeneously broadened InAs QD ensemble. This approach achieves uniform population inversion and broadband rephasing, overcoming the limitations of transform‐limited excitation. Experimentally, we observe a 3.2‐fold enhancement of the PE signal in dense, self‐assembled InAs QDs operating at telecom wavelengths. Numerical simulations based on a two‐level model reproduce the experimentally observed ARP‐induced enhancement, validating the underlying physical mechanism. These results establish ARP as an effective and scalable method for coherent control in THz‐broadened QD ensembles, opening a pathway toward ultrafast and broadband optical quantum memory and communication in the telecom band.

## Graphical abstract

Chirped rephasing pulses satisfying adiabatic conditions enable robust adiabatic rapid passage across a THz‐broadened InAs quantum dot ensemble. This approach provides uniform population inversion and broadband rephasing, overcoming the limitations of transform‐limited excitation. Experimentally, a 3.2‐fold enhancement of the photon‐echo signal is observed, demonstrating a scalable route toward ultrafast and broadband optical quantum memory.

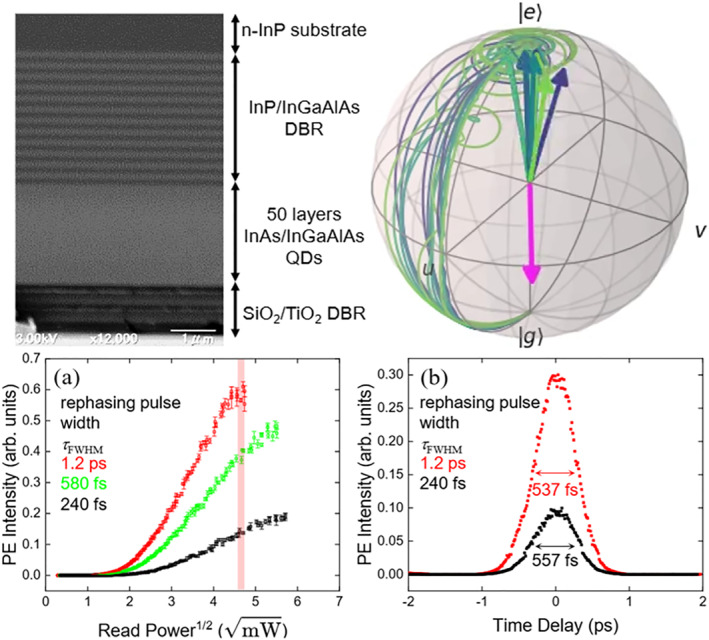

## 1 Introduction

Photon echo (PE) techniques have long served as powerful tools for probing coherence properties and phase relaxation dynamics in inhomogeneously broadened media, offering valuable insights into quantum information science [1]. PE can be understood as a specific form of four‐wave mixing (FWM), a third‐order nonlinear optical process governed by the material's $\chi^{\left(3\right)}$ susceptibility. In this process, macroscopic coherence induced by an initial excitation pulse rapidly dephases due to the inhomogeneous distribution of resonance frequencies across the ensemble. A subsequent rephasing pulse recovers this dephasing, resulting in the emission of a coherent echo at a later time. This rephasing effectively compensates for inhomogeneous broadening and recalls ensemble coherence, providing a direct probe of collective quantum dynamics.

In recent years, PE‐based protocols have attracted renewed attention as promising candidates for optical quantum memory, owing to their intrinsic multimode capacity and compatibility with time‐domain multiplexing [2, 3]. Among various implementations, rare‐earth‐ion‐doped systems such as ${Eu}^{3+}$:$Y_{2}{SiO}_{5}$ crystals and erbium‐doped fibers have emerged as leading platforms [4, 5, 6, 7, 8, 9, 10, 11]. However, their operational bandwidths—typically on the order of GHz—fundamentally limit their suitability for storing sub‐nanosecond optical pulses.

Semiconductor quantum dot (QD) ensembles have attracted significant interest as alternative platforms due to their THz‐scale inhomogeneous broadening and sub‐picosecond excitonic dynamics [12, 13, 14, 15, 16, 17, 18, 19, 20, 21, 22]. In particular, self‐assembled InAs QDs operating at telecom wavelengths offer compatibility with existing fiber‐optic infrastructure and enable direct manipulation using femtosecond optical pulses [23]. Nevertheless, coherent control of such broadband and spatially inhomogeneous ensembles remains challenging. Strong spectral detuning and spatial variation in the excitation field amplitude often result in incomplete rephasing and reduced PE efficiency.

To overcome these limitations, adiabatic rapid passage (ARP) has been explored as a robust technique for quantum state control. ARP employs chirped pulses to achieve population inversion even in the presence of detuning and field inhomogeneities [24, 25, 26, 27], which enables uniform excitation across inhomogeneously broadened ensembles. It has been successfully employed to enhance electromagnetically induced transparency (EIT) in ${Pr}^{3+}$:$Y_{2}{SiO}_{5}$ [28], and to control single or few QDs in nanophotonic structures [29, 30, 31, 32, 33, 34, 35, 36]. Moreover, ARP has also been investigated in nonlinear optical processes, including FWM [37, 38], and has been demonstrated to improve PE efficiency in rare‐earth‐ion‐doped crystal quantum memories [6, 39]. However, in QD‐based studies, ARP has primarily been employed for reliable population inversion at the single‐emitter level, with limited attention to dense QD ensemble coherence. Thus, the potential of ARP for coherent control in dense QD ensembles with THz‐scale inhomogeneous broadening remains largely unexplored.

In this work, we report the first demonstration of ARP‐enhanced PE generation in a self‐assembled InAs QD ensemble operating in the telecom band. By employing chirped optical pulses designed to satisfy ARP conditions across the THz‐scale inhomogeneous broadening—approximately three orders of magnitude wider than in rare‐earth systems—we achieve a pronounced enhancement of ensemble coherence. This enhancement is evidenced by stronger PE signals, despite the need for large chirp bandwidths and robust adiabatic tuning. Numerical simulations based on a two‐level model with realistic system parameters reproduce the observed dynamics and elucidate the underlying mechanisms. Our results establish ARP as a scalable and robust method for broadband coherent control of ultrafast nonlinear processes such as PE and four‐wave mixing. Extending ARP into the femtosecond regime enables manipulation of complex many‐body coherences on unprecedented timescales, opening promising pathways toward ultrafast quantum memory devices and broadband photonic quantum technologies compatible with existing telecom infrastructure.

## 2 Experimental Setup and Theoretical Modeling

### 2.1 Optical Setup and InAs QD Ensemble

**FIGURE 1:**
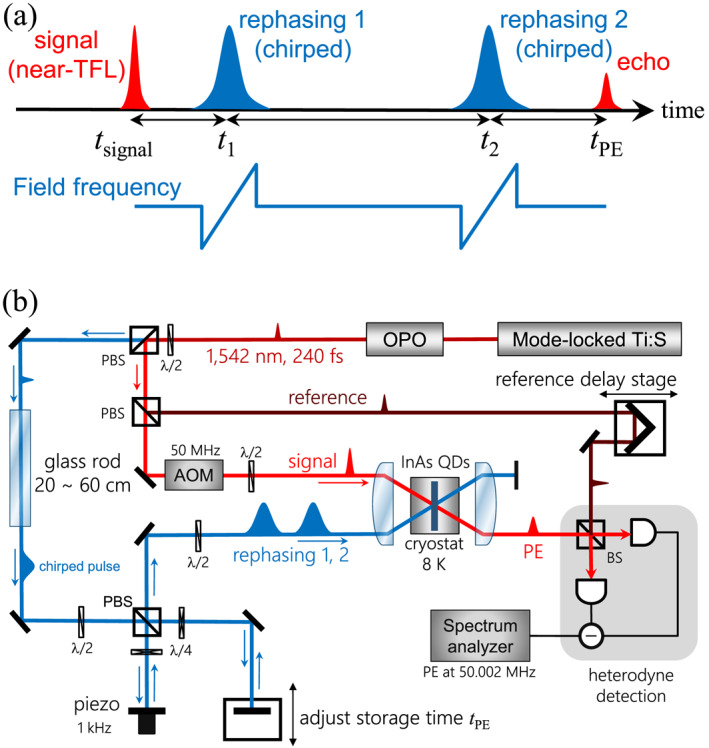
(a) PE pulse sequence of our setup. We applied double rephasing pulses (rephasing 1 and 2). The blue line represents the change in frequency of the rephasing pulses. (b) Optical setup used for PE experiment. λ/2, half wave plate; λ/4, quarter wave plate; BS, half beam splitter; PBS, polarizing beam splitter.

As a method for reading out signals stored in the QD ensemble, we have combined the revival of silenced echo (ROSE) scheme [6] with the ARP technique. In this protocol, two rephasing pulses are used to recover macroscopic coherence, both realized as chirped pulses to induce the ARP effect, as shown in the pulse sequence in Figure 1a. In the ROSE protocol, the echo that would normally be generated after the first rephasing pulse is suppressed because the phase matching condition is intentionally violated, resulting in destructive interference among the emitters and thus preventing any optical emission. The stored macroscopic coherence is instead preserved and subsequently rephased by the second rephasing pulse, leading to the emission of a detectable PE. The temporal relationship between the pulses and the emitted PE is therefore given by:

(1) $$t_{\text{PE}}=t_{\text{signal}}+2\left(t_{2}-t_{1}\right)=t_{\text{signal}}+2t_{21},$$

where $t_{\text{signal}}$, $t_{1}$, $t_{2}$, and $t_{\text{PE}}$ denote the arrival times of the signal, rephasing 1, rephasing 2 and PE pulses with wavevectors $k_{\text{signal}}$, $k_{1}$, $k_{2}$ and $k_{\text{PE}}$, respectively, and $t_{21}=t_{2}-t_{1}$ is the interval between the two rephasing pulses. By adjusting $t_{21}$, the storage time $t_{\text{PE}}$ can be arbitrarily controlled. For simplicity, we set $t_{\text{signal}}=0$. Since the PE must be emitted after the arrival of the second rephasing pulse, the condition $t_{\text{PE}}>t_{2}$ must be satisfied. Applying this condition to Equation (1) yields the requirement $t_{2}>2t_{1}$ for the PE to appear. The directional relationships between the pulses and the resulting PE are given by:

(2) $$k_{\text{PE}}=k_{\text{signal}}+2\left(k_{2}-k_{1}\right).$$

In the case reported here, the two rephasing pulses were co‐aligned $\left(k_{1}=k_{2}\right)$, resulting in $k_{\text{PE}}$ being equal to $k_{\text{signal}}$ and the PE signal being generated in the same direction as the signal pulse.

An overview of our optical setup is depicted in Figure 1b. We employed an optical parametric oscillator (OPO) with a pulse width of 240 fs and a wavelength of 1542 nm, which corresponds to the central resonance wavelength of the QD ensemble. Its spectral width was 3.2 THz, so the pulse width was slightly broader than the transform‐limited (TFL) pulse width (Group Delay Dispersion: $GDD=1.0\times 10^{4}\;{fs}^{2}$). The OPO was pumped by a mode‐locked Ti:Sapphire laser (76.4 MHz, 820 nm).

**FIGURE 2:**
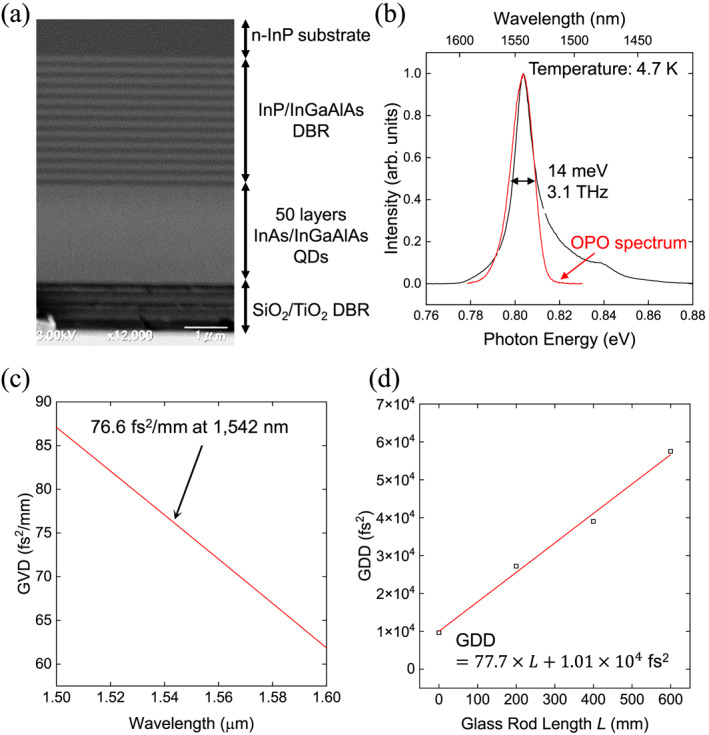
(a) Structure of the InAs QD ensemble integrated with a resonator. (b) Photoluminescence spectrum of the QD ensemble at 4.7 K. The red line indicates the spectrum of the OPO laser. (c) GVD curve of the glass rod based on literature values at room temperature. (d) Experimental result of GDD with different glass rod length. The red solid line represents a fit to the experimental data.

We utilized a self‐assembled InAs QD ensemble embedded in a resonator structure and cooled to 8.2 K in a cryostat for the PE experiments. Figure 2a shows a cross‐sectional SEM image of the sample. The structure consists of 20 pairs of semiconductor distributed Bragg reflectors (InP/InGaAlAs DBRs) grown on an InP(311) substrate, followed by 50 layers of strain‐compensated InAs QDs, and capped with three pairs of dielectric DBRs composed of $SiO_{2}/TiO_{2}$ [40, 41]. The strain‐compensation technique shifts the center resonant frequency into the telecom band. The areal QD density was approximately $2.9\times 10^{12}\;cm^{-2}$, and the average lateral dimensions of the dots were 65 nm along the $\left[\bar{2}33\right]$ crystallographic direction and 50 nm along $\left[01\bar{1}\right]$. Using linearly polarized excitation allowed selective coupling to a specific exciton dipole axis, enabling the system to be treated effectively as a two‐level artificial atom. The semiconductor and dielectric DBRs form a low‐Q (∼100) Fabry–Pérot resonator. This configuration enhances the electric field interacting with the QD ensemble while preserving the THz‐scale inhomogeneous broadening essential for broadband photon‐echo operation. Figure 2b shows the photoluminescence spectrum of the sample, revealing an inhomogeneously broadened linewidth of 3.1 THz. All excitation pulses were polarized along the $\left[\bar{2}33\right]$ axis to selectively excite a single exciton state and maximize the PE signal.

Instead of using a conventional Offner pulse stretcher based on a diffraction grating, we generated positively chirped pulses by varying the pulse width $\tau_{\text{FWHM}}$ (full width at half maximum: FWHM) from 240 fs to 1.2 ps using glass rods (OHARA, S‐NPH3, length L=20∼60 cm). This approach aims to minimize power losses associated with diffraction gratings. Positive chirp was deliberately chosen because previous studies have shown that positively chirped pulses are more efficient for ARP than negatively chirped pulses [42, 43]. The literature value of refractive index of the glass is 1.89, and the group velocity dispersion (GVD) is $76.6,fs^{2}/mm$ at a wavelength of 1542 nm (Figure 2c) [44]. Figure 2d shows the dependence of measured GDD on the length of the glass rod, from which we obtained a GVD value of $77.7\;fs^{2}/mm$, which is in good agreement with the literature value. The PE signal was measured via heterodyne detection using a balanced photodetector, and the temporal waveform was obtained by scanning the reference delay stage.

### 2.2 Single Pulse ARP Dynamics in a Two‐Level System

In actual InAs QD ensembles, the inhomogeneous broadening extends to the THz range, meaning that QDs detuned by Δ from the center frequency of the rephasing pulse are driven far off resonance and therefore are not efficiently excited. In addition, the spatial intensity profile of the laser beam generally follows a Gaussian distribution, resulting in position‐dependent electric‐field amplitudes across the ensemble. Together, these two factors significantly degrade the storage efficiency of broadband quantum memories.

To understand how ARP behaves under such strongly detuned and spatially varying conditions, we first analyze the ARP dynamics in a two‐level system under resonant excitation as shown in Figure 2b. We consider a linearly chirped Gaussian pulse. The TFL pulse width is denoted by $\tau_{0}$, and the presence of linear chirp causes temporal broadening. The chirped pulse width τ is related to $\tau_{0}$ through:

(3) $$\tau =\tau_{0}\sqrt{1+\frac{{GDD}^{2}}{\tau_{0}^{4}}}.$$

The pulse width τ is related to the FWHM by $\tau_{\text{FWHM}}=2\sqrt{\ln\;2}\;\tau$. GDD is related to the temporal chirp parameter α by:

(4) $$GDD=\frac{\alpha \tau_{0}^{4}}{1+\alpha^{2}\tau_{0}^{4}}.$$

Using the pulse width τ, the electric field of the chirped Gaussian pulse can be written as:

(5) $$E\left(t\right)=E_{0}\;\exp\left[-\frac{t^{2}}{2\tau^{2}}-i\omega_{0}t-i\frac{\alpha }{2}t^{2}\right],$$

where $E_{0}$ is the peak amplitude and $\omega_{0}$ is the center frequency.

The time evolution of the quantum state R of each QD is described by the Bloch equation:

(6) $$\frac{\partial }{\partial t}R=\Omega \times R,$$

where Ω is the torque vector. Here, we use the rotating wave approximation (RWA), which allows the fast optical oscillations to be neglected. The torque vector Ω and the pulse area Θ of the Bloch vector are expressed as:

(7) $$\Omega =\left(\begin{array}{c} \frac{2\mu E}{\hbar }\\ 0\\ \Delta -\alpha t \end{array}\right),$$

(8) $$\Theta =\int \sqrt{{\left(\frac{2\mu E}{\hbar }\right)}^{2}+\Delta^{2}}\;dt,$$

where μ is the dipole moment of the QD ensemble, Δ denotes the detuning of each QD from the center frequency of the rephasing pulse, and α represents the linear temporal chirp. Since α=0 when the rephasing pulse is TFL, it can be seen that both the torque vector and the pulse area are affected by the detuning and the inhomogeneous distribution of the electric field.

On the other hand, if the frequency variation of the chirped pulse is sufficiently large, the torque vector gradually moves slowly from one pole to the other, and every Bloch vector adiabatically follows this motion. As a result, ARP enables robust control over variations in Δ and electric field amplitude E. Applying this technique to QD ensembles is expected to address some of the challenges currently faced with these systems. To compare the experimental results and theoretical values, we have performed numerical simulations of both population inversion and PE protocol. The Hamiltonian when a chirped pulse is applied can be shown as follows:

(9) $$H\left(t\right)=\left(\begin{array}{cc} \frac{\hbar \left(\Delta -\alpha t\right)}{2} & -\mu E\\ -\mu E & -\frac{\hbar \left(\Delta -\alpha t\right)}{2} \end{array}\right).$$

In our simulation, we used the density matrix obtained from the Lindblad equation:

(10) $$\begin{array}{cc} \frac{\partial }{\partial t}\rho_{\Delta ,E_{0}} & =-\frac{i}{\hbar }\left[H,\rho_{\Delta ,E_{0}}\right]\\ & +\sum_{k}\left(C_{k}\rho_{\Delta ,E_{0}}C_{k}^{†}-\frac{1}{2}\left\{C_{k}^{†}C_{k},\rho_{\Delta ,E_{0}}\right\}\right) \end{array}$$

where the collapse operators $C_{k}$ are defined using the relaxation constants:

(11) $$\Gamma_{1}=\frac{1}{T_{1}},\;\Gamma_{2}=\frac{1}{T_{2}},\;\Gamma_{\phi }=\Gamma_{2}-\frac{\Gamma_{1}}{2}$$

as follows:

(12) $$C_{1}=\sqrt{\Gamma_{1}}\sigma_{-},\;C_{2}=\sqrt{\frac{\Gamma_{\phi }}{2}}\sigma_{z}.$$

$T_{1}$ and $T_{2}$ are the population relaxation time and the dephasing time, respectively. We then calculated the time evolution of the Hamiltonian using the Python module QuTiP [45], with the simulation parameters summarized in Table 1.

**TABLE 1:** Parameters used in the simulation of single π rotation and PE.

Parameter	Value
Rephasing pulse diameter	200μm
Signal pulse diameter	100μm
Transition dipole moment μ	57Debye
Population relaxation time $T_{1}$	1ns
Dephasing time $T_{2}$	600ps
Initial condition (single π rotation)	|g〉
Initial condition (PE)	$\frac{1}{\sqrt{2}}\left(|g\rangle +|e\rangle \right)$

*Note:* The values are chosen based on experimental measurements to ensure consistency between simulation and experiment [46].

Under our experimental conditions, more than $10^{7}$ QDs are simultaneously irradiated, which justifies treating the distributions over detuning and spatial position as continuous functions. We assume that the center frequency of the QD ensemble is matched to the frequency of the rephasing pulse, and model the inhomogeneous broadening as a Gaussian distribution in the detuning Δ of individual QDs:

(13) $$A\left(\Delta \right)=\frac{1}{\sigma \sqrt{2\pi }}\exp\left(-\frac{\Delta^{2}}{2\sigma^{2}}\right),\;\sigma =\frac{\Delta_{\text{FWHM}}}{2\sqrt{2\;\ln\;2}},$$

where $\Delta_{\text{FWHM}}$ denotes the FWHM of the spectral distribution. The spatial variation of the electric field is modeled by a two‐dimensional Gaussian beam profile:

(14) $$E\left(r\right)=E_{0}\;\exp\left(-\frac{r^{2}}{w_{0}^{2}}\right),$$

where $w_{0}$ is the beam waist.

Taking into account both the spectral and spatial inhomogeneities, the total expectation value of the system is obtained by integrating over all detunings Δ and radial positions r in the transverse plane:

(15) $$\rho_{\text{total}}\left(t\right)=\int_{-\infty }^{\infty }\int_{0}^{\infty }2\pi r\;A\left(\Delta \right)\;\rho_{\Delta ,E\left(r\right)}\left(t\right)\;dr\;d\Delta .$$

**FIGURE 3:**
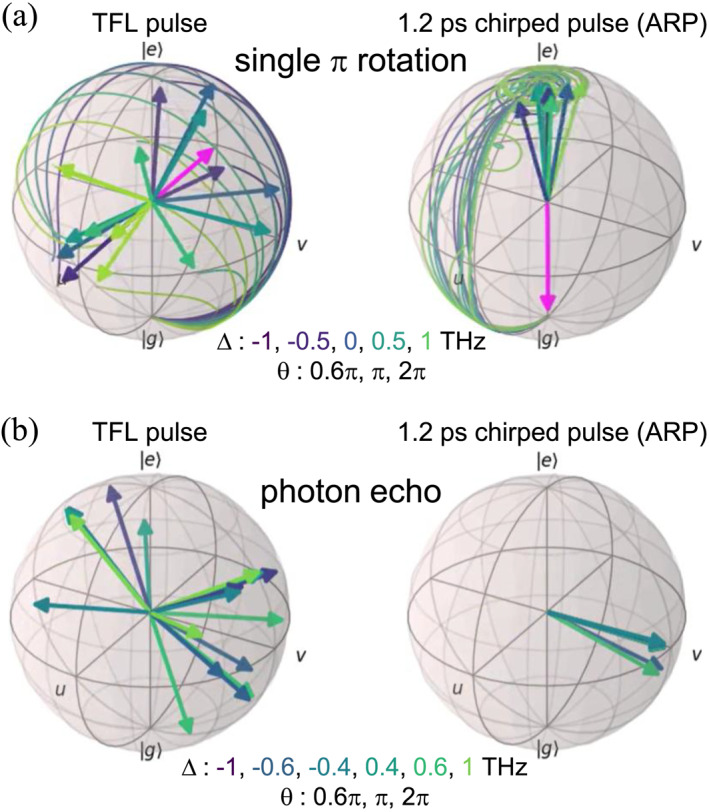
(a) Comparison of final Bloch vector states in population inversion for single TFL and 1.2 ps chirped pulse (ARP) excitation. The magenta arrows represent the final positions of the torque vectors, whereas the solid lines trace the trajectories of the Bloch vectors. The color of each Bloch vector corresponds to its respective detuning. (b) Comparison of final Bloch vector states in PE for TFL and ARP rephasings, starting from the superposition state (v in this figure) as the initial condition.

Figure 3a shows the population inversion simulation results with different detuning values Δ=0,±0.5,±1THz and pulse areas (electric field) E=0.6π,π,2π. These Δ values reflect the typical spectral inhomogeneous broadening in self‐assembled InAs QD ensembles. Compared to the case with the TFL pulse, almost all Bloch vectors are driven to the excited state when a chirped pulse is applied. This result confirms that the ARP technique is effective for achieving robust control even in materials with THz‐range inhomogeneous broadening. For the full motion of the Bloch vectors, see Video S1.

### 2.3 ARP‐Enhanced Photon Echo

Although the principle of ARP is similar to that of conventional population inversion, a key distinction in PE scheme is that the initial quantum state is a coherent superposition, and two chirped pulses are applied to achieve rephasing. In our PE simulations, we employed the time‐dependent Hamiltonian described in Equation (9), which explicitly includes all three optical pulses: the signal pulse, the first rephasing pulse, and the second rephasing pulse. The chirp parameter α was adjusted for each pulse to match the experimentally measured pulse durations. During intervals when no pulse was present, the system evolved under the free precession Hamiltonian given by:

(16) $$H_{\text{free}}=\left(\begin{array}{cc} \frac{\hbar \Delta }{2} & 0\\ 0 & -\frac{\hbar \Delta }{2} \end{array}\right).$$

The total Hamiltonian $H_{\text{total}}\left(t\right)$ is defined as

(17) $$H_{\text{total}}\left(t\right)=\left\{\begin{array}{cc} H_{\text{signal}},\; & -3\tau_{\text{signal}}\leq t<3\tau_{\text{signal}},\\ H_{\text{reph.}1},\; & t_{1}-3\tau_{1}\leq t<t_{1}+3\tau_{1},\\ H_{\text{reph.}2},\; & t_{2}-3\tau_{2}\leq t<t_{2}+3\tau_{2},\\ H_{\text{free}},\; & \text{otherwise}, \end{array}\right.$$

where $H_{\text{signal}}$, $H_{\text{reph.}1}$, $H_{\text{reph.}2}$ correspond to the Hamiltonians for the signal, rephasing 1, and rephasing 2 pulses, respectively.

The parameters $\tau_{\text{signal}}$, $\tau_{1}$, and $\tau_{2}$ represent the temporal standard deviations of each pulse in Equation (3), and the cutoff of ±3τ ensures numerical accuracy while maintaining physical relevance. The PE signal was finally obtained by solving the Lindblad equation given in Equation (10).

Figure 3b illustrates the final states of the PE protocol for Bloch vectors with different detunings Δ=±1,±0.6,±0.4THz and pulse areas Θ=0.6π,π,2π. For a simple demonstration, the pulse timings were set to $t_{1}=1\;ps$, $t_{2}=7\;ps$, and $t_{\text{PE}}=12\;ps$. For TFL rephasing pulses, the final states show significant dispersion, leading to weaker macroscopic coherence and a reduced PE signal. In contrast, with 1.2 ps chirped pulses (ARP), the Bloch vectors are nearly aligned, restoring macroscopic coherence. This demonstrates that even when starting from a superposition state, the application of two chirped rephasing pulses provides robust control against variations in THz‐scale detuning Δ and pulse area Θ. The full Bloch vector dynamics are provided in Video S2.

## 3 Results and Discussion

### 3.1 Simulation Results of ARP‐Enhanced Photon Echo

**FIGURE 4:**
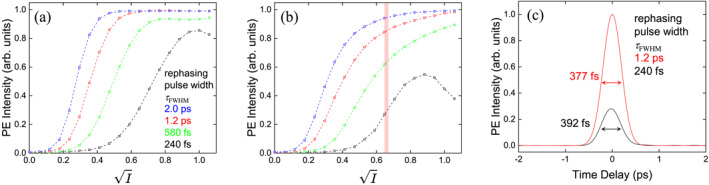
Simulation results showing the dependence of the PE intensity on the square root of the average rephasing pulse intensity $\sqrt{I}$ for (a) a flat‐top and (b) a Gaussian electric field profile. I is normalized such that $\sqrt{I}=1$ corresponds to a pulse area of π for a TFL pulse. The red shaded region indicates the pulse intensity range used in the PE waveform measurement experiment. PE waveforms corresponding to $\sqrt{I}=0.65$, indicated by the red region in (b), are shown in (c).

Figure 4a shows the dependence of the PE intensity on the square root of the average rephasing pulse intensity $\sqrt{I}$ for spatial flat‐top pulses with a uniform transverse intensity profile. Here, I is normalized such that $\sqrt{I}=1$ corresponds to a π‐pulse for a TFL pulse. We simulated four rephasing pulses with different GDD values: $1.0\times 10^{4}$, $3.0\times 10^{4}$, $6.3\times 10^{4}$, and $1.0\times 10^{5}\;fs^{2}$, corresponding to pulse widths $\tau_{\text{FWHM}}$ of 240 fs, 580 fs, 1.2 ps, and 2.0 ps, respectively. As shown in Figure 4a, pronounced Rabi oscillations appear for near‐TFL pulses, but they disappear as the chirp increases, leading to a saturation of the PE intensity. These results demonstrate that ARP suppresses the pulse area dependence and enables robust rephasing in the femtosecond PE process.

For example, with $\tau_{\text{FWHM}}=580$ fs, the maximum PE intensity reaches 93% of the ideal value, whereas for pulses longer than 1.2 ps it recovers the full intensity. This behavior is consistent with the fact that the frequency sweep range, 2ατ, reaches the inhomogeneous width of our QD ensemble, 3.1THz, for GDD $\alpha^{\prime }\geq 4.0\times 10^{4}\;fs^{2}$, with a rephasing pulse width of $\tau_{\text{FWHM}}=670\;fs$. Notably, ARP provides significant enhancement at relatively low intensities, where conventional TFL rephasing fails.

Figure 4b presents analogous simulations for rephasing pulses with a Gaussian spatial profile, chosen to match experimental beam sizes (100 μm for the signal and 200 μm for the rephasing pulse). Compared to the flat‐top case, the PE intensity is reduced by spatial averaging, yet robust rephasing re‐emerges at higher intensities. The corresponding temporal waveforms are shown in Figure 4c. At $\sqrt{I}=0.65$ (red region in Figure 4b), the simulated PE widths are 392 fs without ARP and 377 fs with ARP, indicating that ARP enhances efficiency without degrading temporal bandwidth. The shortening of the PE pulse with ARP is due to contributions from QDs with large Δ, which broaden the PE spectrum. The calculated signal enhancement factor is 3.56. Taken together, these simulations predict that ARP not only mitigates sensitivity to pulse area and spatial inhomogeneity but also preserves sub‐picosecond bandwidth, providing a viable route to efficient PE generation in broadband, inhomogeneous systems.

### 3.2 Experimental Results and Comparison With Theoretical Model

We next tested these predictions experimentally, measuring the storage‐time and pulse area dependence of PE signals in a QD ensemble. In this experiment, we fixed the timing of the first rephasing pulse at t=35 ps and varied the timing of the second rephasing pulse to alter the storage time.

**FIGURE 5:**
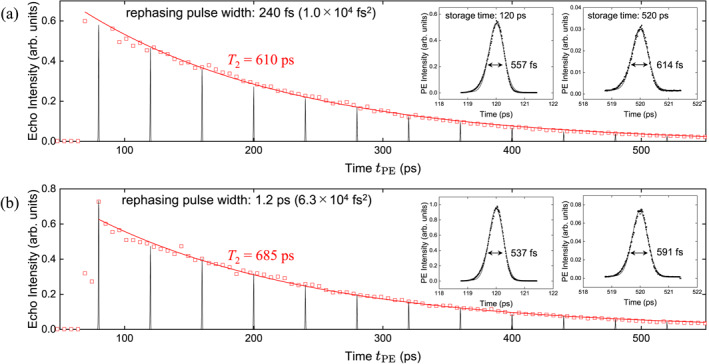
Storage time $t_{\text{PE}}$ dependence of PE intensity (a) without and (b) with ARP. Red squares denote peak PE intensity, red solid lines are exponential fits, and black solid lines are PE waveforms. Insets show expanded PE traces at 120 and 520 ps.

Figure 5 compares the storage‐time dependence of the PE intensity using 240 fs near‐TFL rephasing pulses ($1.0\times 10^{4}$ ${\text{fs}}^{2}$) and 1.2 ps chirped pulses ($6.3\times 10^{4}$ ${\text{fs}}^{2}$), both with an average power of 22.4 mW. The absence of PE signals below 70 ps storage time is due to the time range in which PE does not occur, as determined by the $t_{1}$ value. Exponential fits of the form $\exp\left(-4t/T_{2}\right)$ yield dephasing times $T_{2}$ of 610 and 685 ps, respectively. These values are comparable to those reported for single InAs QDs in previous studies [23, 47]. The modest extension of $T_{2}$ (610 → 685 ps) primarily reflects reduced excitation‐induced dephasing due to the chirp‐induced reduction in peak power, while ARP ensures robust rephasing across detuning variations. Under weak excitation, $T_{2}$ extended to approximately 1.5 ns. The application of ARP enables robust control even at lower peak powers, resulting in a less pronounced coherence decay compared to high‐peak‐power TFL pulses. Insets show representative PE waveforms, from which the actual widths were estimated using the original OPO pulse width. At $t_{\text{PE}}=120$ ps, the PE widths were 557 fs (TFL) and 537 fs (ARP), whereas at $t_{\text{PE}}=520$ ps they broadened to 614 and 591 fs, respectively. The shorter PE pulse width observed with ARP reflects robust control over frequency detuning, whereas the broadening at longer storage times arises from ensemble decoherence. Extrapolation to $t_{\text{PE}}=0$ indicates a maximum PE generation efficiency of $4.2\times 10^{-3}$% without ARP and $1.3\times 10^{-2}$% with ARP. The efficiency remains limited due to the small absorption of our InAs QD sample. Improvements could be achieved by increasing absorption via waveguide structures or by enlarging the beam diameter to address more QDs. These approaches would increase the overall PE efficiency while preserving the enhancement effect of ARP, suggesting that ARP will continue to be a powerful tool in future experiments.

**FIGURE 6:**
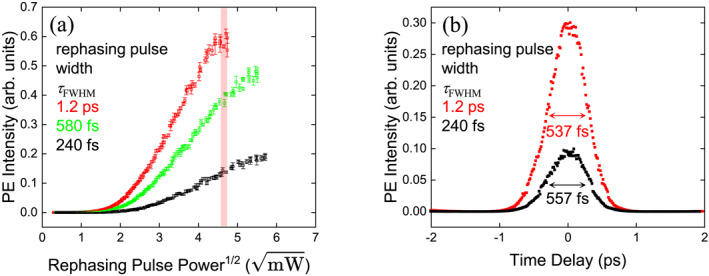
(a) Experimental pulse area dependence of PE intensity for different chirp amounts, where the horizontal axis is proportional to $\sqrt{I}$. (b) PE waveforms corresponding to the red regions in (a). Experimental waveforms were obtained by heterodyne detection. The quoted widths were estimated from Gaussian fits after deconvolution.

The dependence of the PE intensity on the rephasing pulse power is summarized in Figure 6a, where the horizontal axis is proportional to $\sqrt{I}$. The experimental results closely follow the simulation trends shown in Figure 4b, confirming that ARP governs the rephasing dynamics. Temporal waveforms at equal rephasing pulse power (22.4 mW, red regions) are presented in Figure 6b, demonstrating a 3.2‐fold enhancement in PE intensity with ARP. The measured widths were 557 fs without ARP and 537 fs with ARP, consistent with the preservation of ultrafast bandwidth. This enhancement factor is comparable to those reported for rare‐earth‐ion‐doped crystals, underscoring the robustness of ARP in highly inhomogeneous semiconductor ensembles. The slight discrepancy between experiment and simulation is attributed to residual chirp in the echo and spectral filtering by the resonator. The experiments cover only the low pulse area regime, as higher pulse areas are limited by available laser power and by the reduced number of QDs when focusing the beam. Nevertheless, the observed enhancement clearly demonstrates the effectiveness of ARP in dense InAs QD ensembles and provides a solid foundation for future investigations at higher pulse areas.

Overall, our combined simulations and experiments show that ARP enables reliable PE rephasing in broadband, THz‐scale inhomogeneous ensembles. Quantitatively, ARP increased the PE efficiency by a factor of 3.2 while preserving femtosecond temporal widths. These results establish ARP as a practical strategy for coherent control of QD ensembles, opening new opportunities for broadband quantum memory and ultrafast quantum information processing compatible with telecommunication wavelengths.

## 4 Summary

We have demonstrated, for the first time, the application of ARP to a highly inhomogeneous quantum system in the THz spectral regime: a self‐assembled InAs QD ensemble integrated with a resonator. By employing chirped optical pulses satisfying adiabatic conditions across the ensemble's ultrabroad linewidth, we achieved robust quantum control despite spectral detuning and field inhomogeneity. Experimentally, this resulted in up to a 3.2‐fold enhancement in PE intensity compared to TFL pulses, in agreement with numerical simulations. Despite the THz‐scale inhomogeneous broadening, we observe this level of enhancement comparable to that in rare‐earth ion‐doped crystals. Furthermore, the PE efficiency is expected to improve with optimized QD samples, and the combination of THz‐bandwidth excitation with ARP enhancement demonstrates the significant potential of this approach. Simulations confirmed that, even with chirped pulses for ARP, the PE temporal width remains in the femtosecond regime. This work provides the first demonstration of ARP at femtosecond timescales, showing that ultrafast pulses can be coherently rephased without broadening. Such control enables manipulation of collective coherence and sub‐picosecond pulse storage in the telecommunication band, paving the way for ultrafast quantum communication and broadband photonic quantum technologies. Future work will focus on optimizing the excitation beam profile and mode matching between the optical field and the QD ensemble. In particular, aligning the resonator's resonance with the QD absorption peak near 1520 nm and employing flat‐top beams with uniform intensity are expected to further enhance PE signal generation, advancing the realization of scalable, high‐speed quantum memory devices and ultrafast nonlinear optical applications.

## Author Contributions

**Yuta Kochi:** conceptualization, methodology, software, validation, formal analysis, investigation, data curation, writing – original draft, visualization, funding acquisition. **Yutaro Kinoshita:** investigation, validation. **Masanari Watanabe:** methodology, investigation, validation. **Ryutaro Ide:** investigation, resources, validation. **Kouichi Akahane:** resources, writing – review and editing. **Junko Ishi‐Hayase:** conceptualization, resources, supervision, project administration, funding acquisition, writing – review and editing. All authors have accepted responsibility for the entire content of this manuscript and approved its submission.

## Supporting information


**Video S1:** ARP on population inversion.

**Video S2:** ARP on photon echo.

## Data Availability

The data that support the findings of this study are available from the corresponding author upon reasonable request.
